# Supplementary material for: A high-resolution mRNA expression time course of embryonic development in zebrafish
Source: eLife. 2017 Nov 16;6:e30860. doi: 10.7554/eLife.30860 (PMC5690287; doi:10.7554/eLife.30860)
Supplement: Supplementary file 6. [file elife-30860-supp6.zip › biolayout-clusters-files/Cluster138-genes.html]

Cluster138


# Cluster138: Genes

| Ensembl ID | Gene Name | Chr | Start | End | Biotype |
| --- | --- | --- | --- | --- | --- |
| ENSDARG00000087206 | ENSDARG00000087206 | 4 | 76623605 | 76624966 | protein\_coding |
| ENSDARG00000098695 | ENSDARG00000098695 | 12 | 46798830 | 46823456 | protein\_coding |
| ENSDARG00000102122 | NEK9 | 17 | 53146447 | 53156285 | protein\_coding |
| ENSDARG00000052652 | fermt1 | 20 | 45871713 | 45908601 | protein\_coding |
| ENSDARG00000007955 | iars | 11 | 1311033 | 1365873 | protein\_coding |
| ENSDARG00000058543 | lama5 | 23 | 9632364 | 9833730 | protein\_coding |
| ENSDARG00000060184 | pkn1a | 1 | 45063144 | 45144372 | protein\_coding |
| ENSDARG00000079253 | plch1 | 18 | 34006922 | 34166562 | protein\_coding |
| ENSDARG00000090230 | tmem108 | 24 | 9603626 | 9732820 | protein\_coding |
